# Development and Evaluation of PLGA Nanoparticle-Loaded Organogel for the Transdermal Delivery of Risperidone

**DOI:** 10.3390/gels8110709

**Published:** 2022-11-02

**Authors:** Naz Dilawar, Tofeeq Ur-Rehman, Kifayat Ullah Shah, Humaira Fatima, Aiyeshah Alhodaib

**Affiliations:** 1 Department of Pharmacy, Quaid-i-Azam University, Islamabad 45320, Pakistan; 2 Department of Physics, College of Science, Qassim University, Buraydah 51452, Saudi Arabia

**Keywords:** PLGA nanoparticles, antidepressants, risperidone nanoparticles, transdermal drug delivery systems, organogel

## Abstract

A transdermal delivery approach may circumvent the limitations associated with the oral use of risperidone (RIS), an atypical antipsychotic drug. The current study focuses on the utilization of poloxamer (pluronic) lecithin organogel (PLO), a suitable transdermal vehicle, and a biodegradable nanoparticulate system of PLGA with the potential to deliver RIS in an efficient way. PLGA nanoparticles were fabricated using different ratios of the polymer and surfactant. The optimization was performed principally on the basis of particle size and entrapment efficiency (EE). The developed PLGA nanoparticles were spherical, sized around 109 nm with negative charge (−9.3 mv) and enhanced drug entrapment efficiency (58%). The in vitro drug release study of lyophilized nanoparticles showed a sustained pattern. Statistical analysis confirmed that there was a significant difference (*p* < 0.05) between the nanoparticle-loaded PLO gel and conventional drug formulations in terms of drug release and ex vivo permeation across rat skin (three-fold). The results confirm enhanced drug release and permeation through the skin at 72 h. Hence, the investigated formulation could be a better alternative to the conventional route for improving patient compliance.

## 1. Introduction

Recently, researchers have been effectively using poloxamer (pluronic) lecithin organogel (PLO), which is a microemulsion-based gel, to deliver both hydrophilic and lipophilic drugs topically or transdermally across the stratum corneum [1]. PLO is generally a yellow opaque preparation comprising isopropyl-(palmitate or myristate) soya lecithin (phospholipids: a penetration enhancer), an organic solvent, poloxamer (a surfactant gel forming agent and solubilizer) and a polar solvent (water) [1,2]. Poloxamer is a block copolymer that exhibits a reversible thermosensitive property that plays a very crucial role in formulation development. It is not only used for drug solubilization but also disrupts the lipid bilayer and acts as a permeation enhancer [3]. Depending on the solubility of the drug, an important aspect is the incorporation of the drug within PLO either by dispersing in the prepared PLO or in any one of the oil and aqueous phases before the mixing of both phases [4]. Some recent studies reveal that the cumulative drug release decreases with the increase in lecithin concentration, possibly due to a highly viscous network-like structure [5].

PLO gels are biocompatible, nonirritating to the skin and odorless and, most importantly, allow the quick drug delivery across the skin. They are also found to be very effective in the moisturizing and revitalizing keratin-like tissues, including hair and fingernails [6,7].

PLO gels are confirmed to be an excellent drug carrier with enhanced penetration by many in vivo studies. Upon the repeated topical application of methimazole to the inner pinna of some healthy cats (suffering from hyperthyroidism), an enhanced penetration of the drug was observed to the systemic circulation in comparison to a single application. The result was assumed to be because resistance to drug penetration was decreased by the exfoliation and inflammation of the stratum corneum caused by lecithin [8]. In another study, a transdermal delivery of fish oils, namely eicosapentaenoic acid (EPA) and docosahexaenoic acid (DHA), was achieved using PLO-based gel by a repeated dosage regimen, where the gel matrix retained a part of these lipophilic molecules. The same study also demonstrated the delivery of ketoprofen using PLO gel while retaining a lesser extent of molecules in its matrix [9].

Giordano and his coworkers demonstrated that a single topical application of ondansetron (a 5-HT receptor antagonist)-loaded PLO gel resulted in a greater pain reduction in human volunteers who were being stimulated with flare and mechanical hyperalgesia by the intradermal capsaicin administration [10]. In order to treat osteoarthritis of the knee and lateral epicondylitis, a diclofenac-loaded gel of PLO reduced the pain to a higher extent. When applied transdermally, acetaminophen-loaded PLO gel was found to be very effective in cancer patients (hospice setting) [11].

Risperidone, a potent inhibitor of serotonin 5-HT_2_ and dopamine receptor D_2_ receptor located in the brain, is an atypical antipsychotic agent, which is largely utilized as the first line of therapy in psychosis [12]. Risperidone is a BCS class II drug with low solubility and high permeability that exhibits poor oral biopharmaceutical properties [13]. Marketed dosage forms frequently shows low bioavailability due to rapid first pass metabolism, and it produces dose-related side effects, such as extrapyramidal effects (inherent with antipsychotics) and orthostatic hypotension [14]. Orthostatic hypotension is a common side effect of all atypical antipsychotics, including risperidone. It is caused by the blockage of the alpha-1 adrenoceptor [15]. Therefore, patients usually show noncompliance due to these effects.

The PLO-based delivery system enhances the permeation profile of the therapeutic agent through the layers of skin as it imparts hydrating properties to the skin, which is one of the prerequisites for drug penetration. Because patients must cope with the problem of side effects associated with orally delivered risperidone, doses of the therapeutic agent must be kept at minimum. For this reason, we decided to focus on the design of a nanocarrier system that will improve the associated limitations. Moreover, we utilize the administration route that is comparatively a more suitable alternative to oral delivery. Finally, the noncompliance factor was addressed with the successful sustained delivery of the therapeutic agent.

## 2. Results

### 2.1. Drug–Carrier Compatibility Profiling

**Figure 1:**
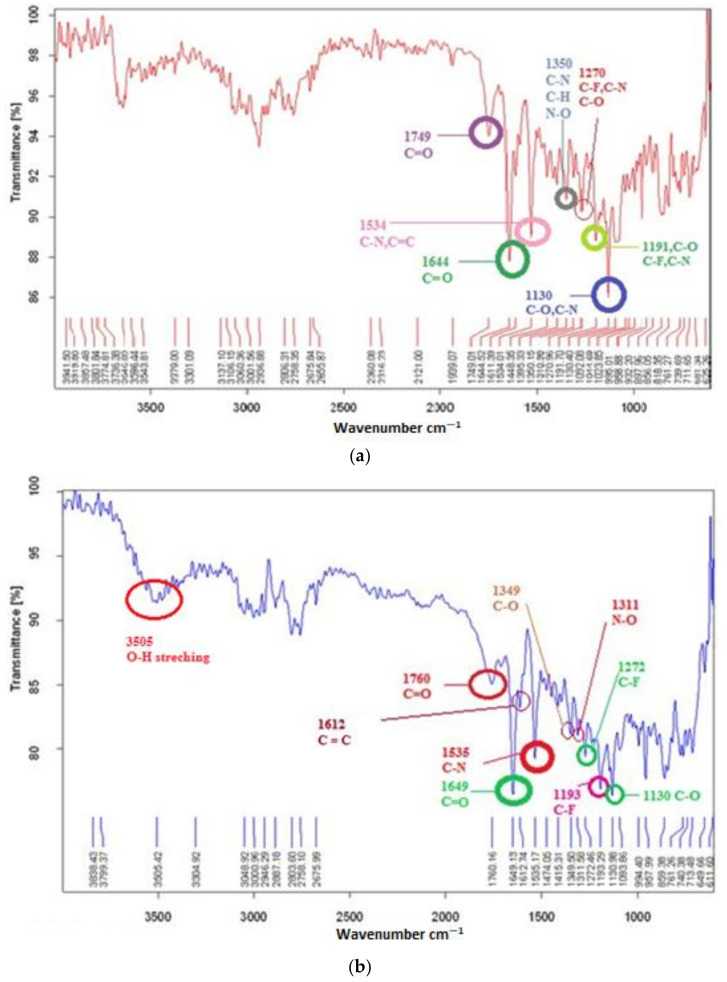

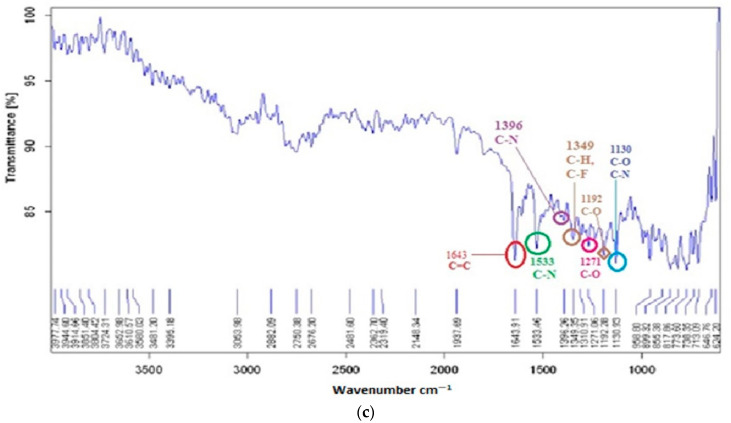
FTIR spectra for (**a**) physical mixture, (**b**) NP formulation and (**c**) drug (RIS).

The comparative FTIR spectra of the physical mixture of the drug and the NP formulation are shown in Figure 1. The FTIR spectra of risperidone showed characteristic peaks at 1643 cm^−1^ due to the stretching of functional groups C=O of aromatic ketone and peak at 1533 cm^−1^ due to C=C stretching of the arene ring. The peaks at 1396 cm^−1^, 1349 cm^−1^, 1271 cm^−1^, 1192 cm^−1^ and 1130 cm^−1^ were due to the functional groups of C-N, C-H, C-F and C-O, respectively [16]. In polymer spectra, stretchings at 3564 cm^−1^, 1750 cm^−1^, 1648 cm^−1^, 1612 cm^−1^, 1534 cm^−1^, 1350 cm^−1^ and 1192 cm^−1^ indicated the presence of the functional groups O-H, C=O, C=C, C-N, C-F and C-O, respectively. The FTIR spectra of the final NP formulation also had characteristic peaks of both drug and polymer at 1760 cm^−1^, 1649 cm^−1^, 1535 cm^−1^, 1349 cm^−1^, 1272 cm^−1^, 1193 cm^−1^ and 1130 cm^−1^, proving that there was no significant change in the functional group of the drug during the nanoformulation process.

### 2.2. Fabrication of RIS-Loaded PLGA Nanoparticles

#### 2.2.1. Screening for the Optimized RIS-Loaded PLGA Formulation

Different formulations of RIS-loaded PLGA nanoparticles were screened using various ratios of the formulation ingredients. Details for the ratios along with their effects in the form of particle size and EE are presented in Table 1. Average particle diameters, EE (%) of 123 nm (35%), 109 nm (58%) and 228 nm (47%), were observed by increasing the surfactant to polymer ratio, i.e., D1 = 1:2.24, D2 = 1:1.1, D5 = 1.78:1, respectively.

The concentration of the polymer was increased in formulations D6 and D7 with a surfactant to polymer ratio of 1:1.5–1:2, providing an increased particle size of 205 nm (56% EE) and 294 nm (58% EE), respectively.

Similarly, in the case of formulation D4, as the drug contents were increased in the RIS to polymer ratio of 1:5.6, there was an increase in average particle diameter (168 nm) and a decrease in the % EE (39%), as shown in Table 1. The D3 formulation consisting of a high volume of aqueous phase (O:A ratio was 1:3) decreased the %EE up to 20% with a lower particle diameter of 130 nm.

#### 2.2.2. Screening for the Selected D2 Formulation

The D2 formulation was further evaluated by changing different parameters, including the injection rate of the organic phase, stirring rate of the aqueous media and sonication. These parameters were not determined initially during its preparation and the results are presented in Table 2.

It was observed that, in D2a formulation, a lower stirring rate (100 rpm) increased the particle size (206 nm) and decreased the %EE (34%). In D2e, sonication was employed, which indicated that sonication decreased the particle diameter up to 194 nm. The D2b and D2d formulations had the same particle sizes and %EE at varying rates of injection and stirring.

### 2.3. Particle Size and Size Distribution of Nanoparticles

**Figure 2:**
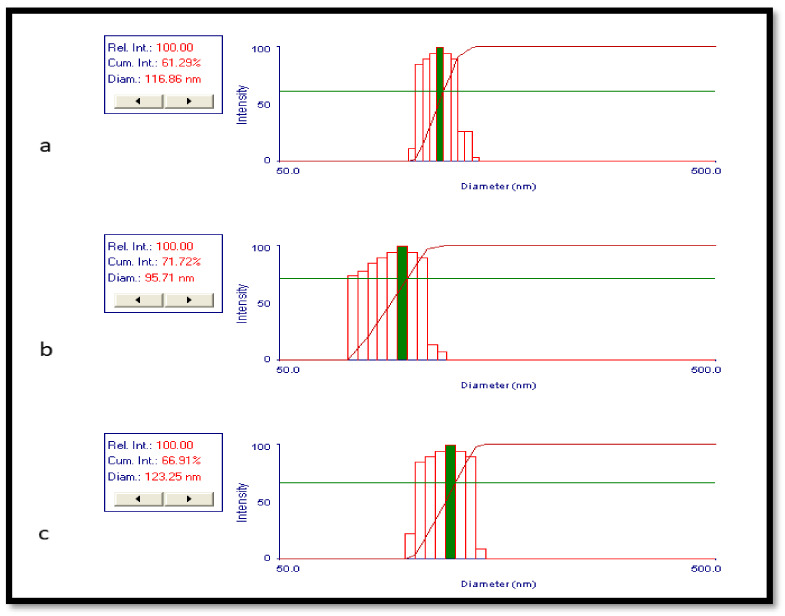
Dynamic light scattering results for particle size and size distribution: (**a**) Blank PLGA NPs and (**b**,**c**) RIS-loaded NPs.

The particle size and size distribution of nanoparticles were evaluated by a dynamic light scattering (DLS) instrument. The mean diameter of the PLGA nanoparticle samples had a variation of 111–294 nm and their PDI was in the range of 0.02–0.69. All results represent mean values of (*n* = 2) ± S.D. The final optimized formulation among the data was D2, which had a smaller diameter (109.48 ± 13.7 nm) and good PDI (0.11 ± 0.01), thus indicating that the D2 nanoparticle solution had a homogeneous distribution (Figure 2).

### 2.4. Zeta Potential

**Figure 3:**
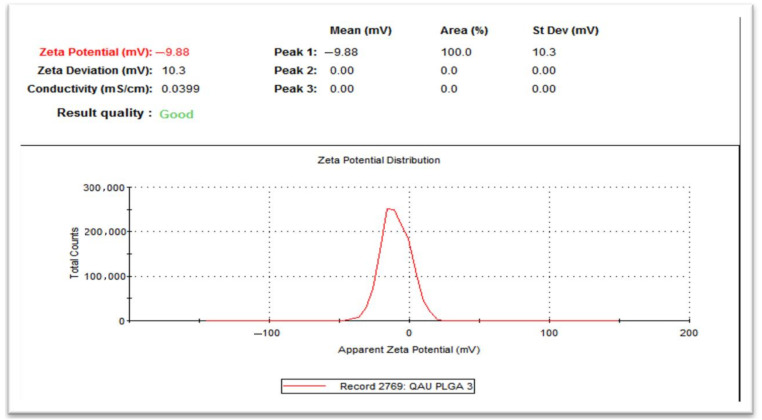
Zeta potential of the final PLGA nanoparticle preparation.

The result of the zeta potential of the final PLGA nanoparticle preparation is presented in Figure 3. The result indicates that it has an average negative charge of −9.3, which can provide steric stability to the particles in the solution.

### 2.5. Determination of Drug-Loading Parameters

The evaluated loading parameters for the prepared PLGA nanoparticles are shown in Table 1, determined with mean ± S.D. (*n* = 3). These parameters mainly depend on the polymer and surfactant ratio. The results indicate that increasing the polymer amount could improve the percentage of drug loading in nanoparticles, but it also increases the diameter of particles.

#### 2.5.1. Entrapment Efficiency (%)

It is an indirect method of determining the total drug content in nanoparticles. It provides information about the drug percentage that is successfully entrapped into NPs. The entrapment efficiency had a variation of 11–58%. The selected optimized preparation (D2) had an optimum EE, i.e., 58.6 ± 0.8%.

#### 2.5.2. Drug Loading (%)

Loading capacity or drug loading refers to the polymer carrying capacity of a drug. It is a direct method of determination of total drug contents from nanoparticles. The obtained range for LC was 6–72%. Our optimized formulation (D2) had an acceptable LC (54.7 ± 0.28%).

#### 2.5.3. Nanoparticle Yield (%)

Nanoparticle yield refers to the percent recovery of nanoparticles during preparation. NP yield for the D2 formulation was found to be 25% and all the other samples had a percent yield in the range of 18–40%.

#### 2.5.4. Surface Morphological Analysis

**Figure 4:**
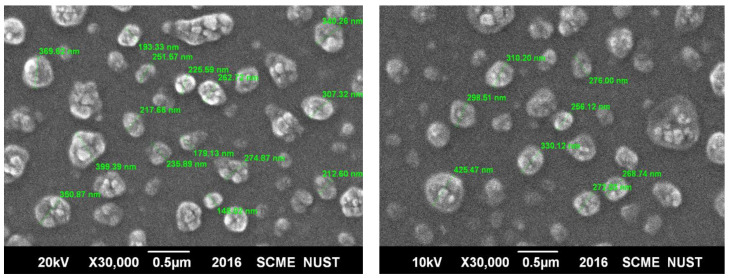
SEM image of RIS-loaded PLGA formulation at different resolutions along with their particle size at 30,000×.

The external surface of NPs was analyzed by SEM. SEM images revealed the spherical shape of PLGA nanoparticles. It also confirmed that the size of the particles was in the range of 180–300 nm. The increase up to 300 nm could be due to the cryoprotectant used during the lyophilization process. The following results (Figure 4) are representative of drug-loaded NP formulations at different resolutions, confirming that the drug was successfully encapsulated in the polymer.

### 2.6. Powder X-ray Diffractometer (PXRD) Analysis

**Figure 5:**
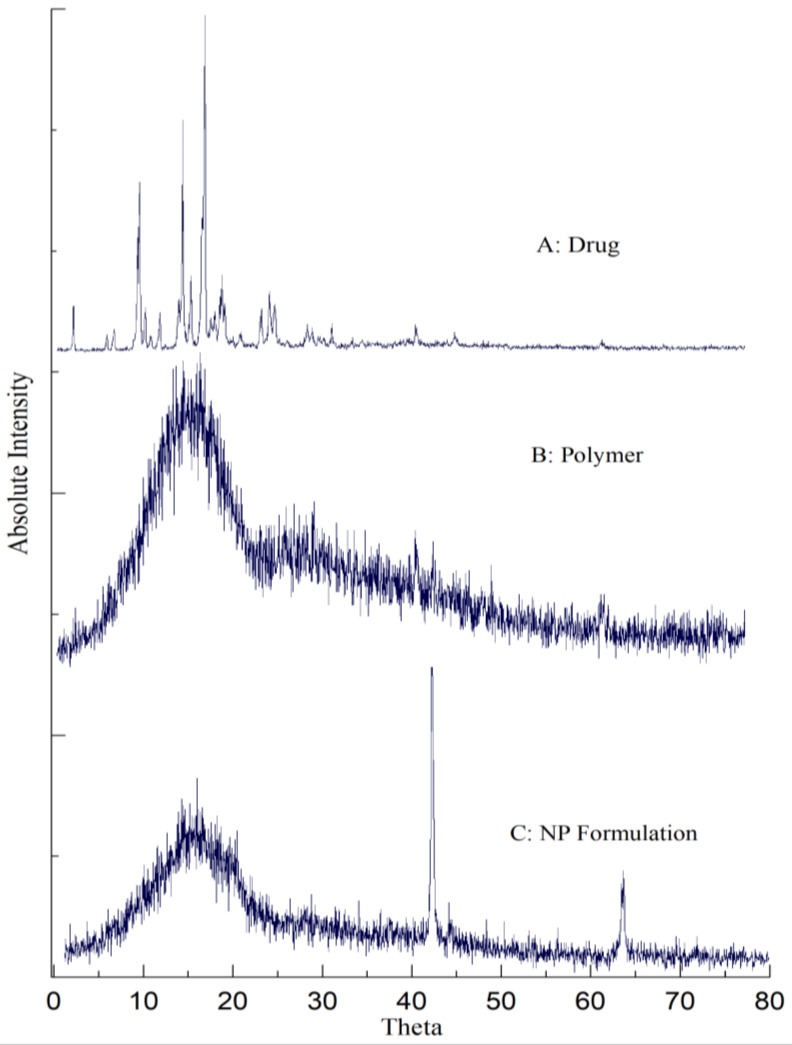
PXRD spectra for (**A**) pure drug, (**B**) polymer and (**C**) RIS-loaded NPs.

The PXRD patterns for risperidone-loaded nanoparticles are shown in Figure 5. The diffractogram of pure risperidone (A) showed characteristic sharp peaks at 2θ values of 14, 18, 19, 21, 23, and 28 (approximately), which exhibits its crystalline nature. The diffractogram of PLGA (B) showed no intense peak, indicating its amorphous nature. The intrinsic peaks corresponding to risperidone disappeared in the diffraction pattern of the final nanoparticles formulation (C), confirming its amorphic nature.

### 2.7. In Vitro Drug Release Studies for NPs

**Figure 6:**
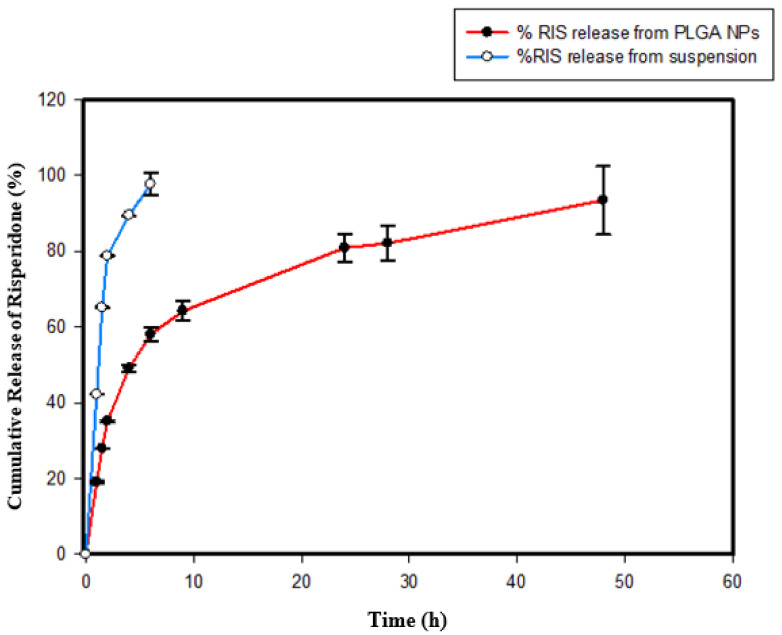
In vitro release profile of RIS from PLGA NPs and simple suspension in PBS (pH = 7.4, with 0.25% tween 80) at 37 °C. Error bars represent standard deviation (*n* = 3).

The in vitro release study was conducted for the release of RIS from PLGA NPs and simple drug suspension (as the control) in PBS (pH = 7.4, with 0.025% Tween 80) at 37 °C (Figure 6). The RIS release profile showed that about 97% ± 2.9 of the drug was diffused in 6 h from the control as compared to the NPs, which showed 57% ± 1.8 release at the same time. NPs showed a biphasic release pattern in the graph with an initial burst release for 2 h followed by a sustained release for 48 h. As the control formulation showed a faster release rate, the difference was considered significant with a low *p*-value of 0.01 (*t*-test).

### 2.8. Characterization of PLO

The individual poloxamer gels with P407 concentrations of 21 and 23 (*w*/*w*%) were transparent and smooth in consistency, while PLO gels were beige, viscous and gritty in nature. When both were mixed, a smooth off-white gel formulation was observed at room temperature. The pH of PLO gels was recorded in the range of 6.3–6.7.

The PLO4 formulation containing the maximum concentration of poloxamer (23%) and lecithin (20%) had a minimum T_gel_ of 23°C. The gelation transition time for poloxamer gel decreased when it was combined with lecithin. The gel transition time for PLO4 was noted to be the lowest (30 s) of all the formulations. The PLO4 formulation had a gel strength of 60 s as compared to other PLO formulations. The viscosity of the combined gel (PLO4) was 3000 poise as compared to the control formulation (P gel 23%), i.e., 2300 poise.

### 2.9. In Vitro Drug Release Studies for PLO

**Figure 7:**
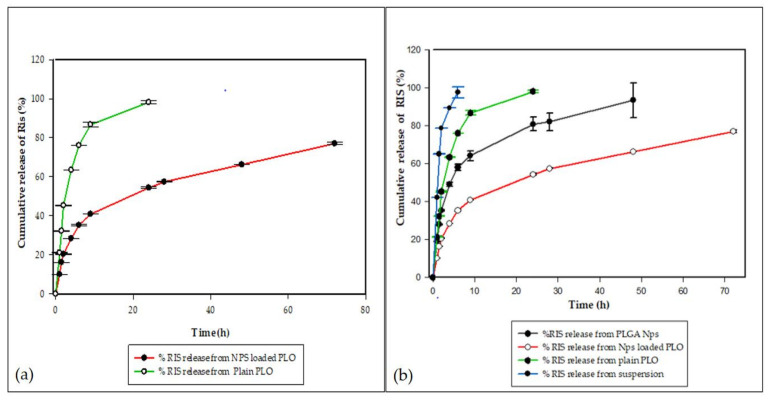
In vitro drug release comparative profiles for (**a**) RIS from NP-loaded PLO and plain PLO in PBS (pH = 7.4, 0.25% tween 80) and (**b**) RIS from simple PLGA NPs, NP-loaded PLO, plain PLO and simple suspension with mean ± S.D. Error bars shows standard deviation (*n* = 3).

The in vitro release profile of RIS for optimized NP-loaded PLO, in comparison with the plain PLO (as the control) in PBS (pH = 7.4, 0.25% tween 80), is presented in Figure 7. The RIS diffusion profile results were constructed as average percent cumulative release versus time by using the sigma plot software. These studies were carried out for 72 h but comparable only for 24 h because about 98% of the drug was released from plain gel in 24 h. In graph 7(a), NP-loaded PLO’s comparison with the control gel indicates that, in the first hour, the control gel showed a 21% ± 0.22 drug release, while the NP-loaded PLO showed only a 10% ± 0.26, 98% drug release, and after 24 h, the control gel provided a drug release of 98% ± 0.64, while the NP-loaded PLO showed only a 54% ± 0.42 drug release.

Graph (b) in Figure 7 represents the comparative release profile of RIS from simple NPs, NP-loaded PLO, plain PLO and from the simple suspension. The results indicate the successful entrapment of RIS in PLGA and that NPs sustained a drug release of 93% ± 9.0 for 48 h. The initial burst release associated with PLGA NPs for 2 h (35% ± 0.2 release) was retarded by PLO consistency to such an extent that only (20% ± 0.02) drug release was observed for 2 h. The NP-loaded organogel showed a total release of 77% ± 0.55 for 72 h as compared to the NPs. When the drug is dispersed in plain PLO, it is solubilized and provides 98% ± 0.64 release in 24 h, which is a much faster drug release as compared to the NP-loaded PLO; therefore, a significant difference among their releases from the different formulations was observed, with a *p*-value of 0.01 (ANOVA).

### 2.10. Best Fit Kinetics Model for the Release Studies

In vitro release data of RIS from PLGA-NPs and NP-loaded PLO were fitted to different equations and kinetic models to explain the release kinetics of the drug from these formulations by using DD solver.

**Figure 8:**
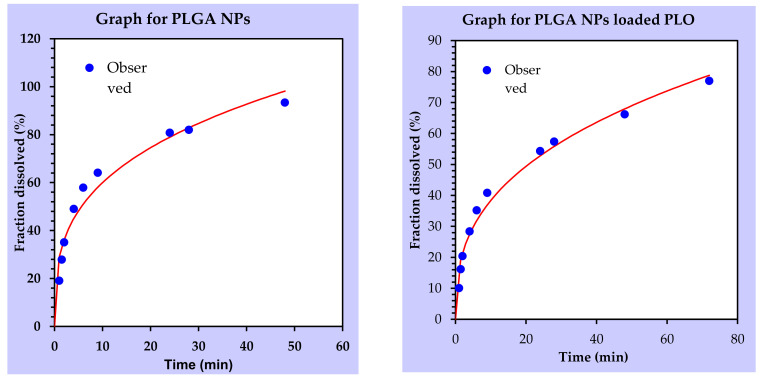
Best fitness graphs of the Korsmeyer–Peppas model of PLGA NPs and NP-loaded PLO obtained with a DD solver.

The highest value of R^2^ indicates the best fitness of that model to drug release. Different models were applied to release profiles, i.e., zero-order, first order, Higuchi’s model, Korsmeyer–Peppas model and Hixson–Crowell model, but both PLGA-NPS and NP-loaded PLO showed the highest coefficient value (R^2^) for Korsmeyer–Peppas model and confirmed that drug release is mainly due to a Fickian diffusion process as *n* = 0.3. The NPs and PLO graphs for Korsmeyer–Peppas models are shown in Figure 8.

### 2.11. Ex Vivo Permeation Studies

**Figure 9:**
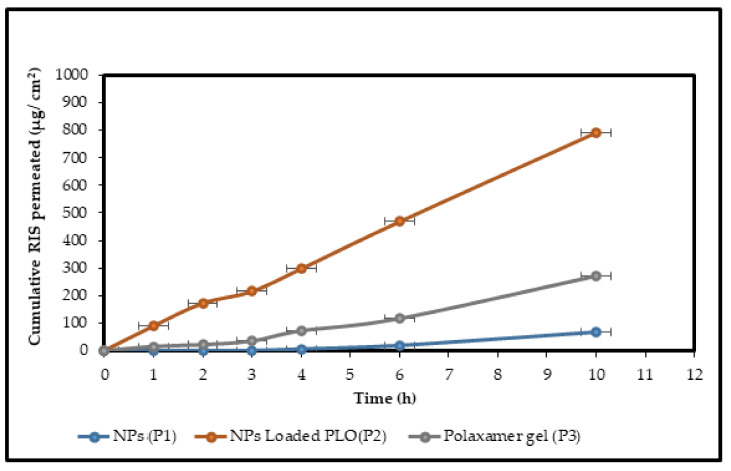
Graph represents the cumulative amount of RIS permeated across rat skin in the cases of NPs (P1), NP-loaded PLO (P2) and P gel (P3). Mean ± S.D. (*n* = 3).

The permeation profiles (Figure 9) of the drug-loaded NPs, optimized NP-loaded PLO (as treatment) and poloxamer gel (23% as the control) across rat skin were obtained using a horizontal Franz cell diffusion apparatus. The permeability values are shown in Table 3. The skin penetration flux for simple NPs (P1, no permeation enhancer) is too low, with a value of 12.7 ± 5.2 µg/cm^2^/h, but when these nanoparticles were loaded into the poloxamer gel (having non-ionic surfactant as a permeation enhancer), their flux increased to a value of 31.53 ± 0.04 µg/cm^2^/h. The flux value for NP-loaded PLO is 82.67 ± 2.8 µg/cm^2^/h, which is three times higher than the control gel (P3) due to the synergistic effect of lecithin with poloxamer in PLO. So, the penetration rate of P2 is statistically significant (*p* < 0.04; *t*-test) in comparison with P2 and P1 (*p* < 0.01; *t*-test).

The magnitude of RIS flux across SC was noted as enhancement ratio (ER) as shown in Table 3 for NP-loaded PLO (P2). The ER ratio indicates that the addition of lecithin increased the enhancement factor by 2.6-fold.

The combination of poloxamer and lecithin provided a synergistic effect on drug permeation [17]. The observed enhanced penetration of methimazole in systemic circulation after a single topical application revealed that it was due to exfoliation and interaction of lecithin.

## 3. Discussion

The spectra for risperidone showed the same characteristic peaks as previously mentioned [16]. FTIR spectroscopy revealed that no extra functional group and bond were observed in the NP forms, and it was evident that RIS was intact and there were no pronounced chemical interactions.

Risperidone was successfully fabricated as nanoparticles applying the nano-precipitation method [18], where the impact of composition parameters, such as surfactant concentration, polymer concentration, drug contents, organic to aqueous phase volume ratio and drug to polymer ratio, was assessed on particle size and EE (Table 1).

Increasing the surfactant concentration from 0.25% to 1% (surfactant: polymer 1 = 1:2.24, 1:1.1, and 1.78:1) showed a nonlinear relationship with an average particle diameter (123, 109 and 228 nm, respectively) and EE (35%, 58% and 47%, respectively). Mainardes and Evangelista investigated the effect of surfactant concentration on the particle size of PLGA and showed that the increase in PVA concentration of 0.15–0.7% in the aqueous phase resulted in a lowering of the particle size of 345–242 nm [19]. Budhian et al. observed the non-linear trend of the particle size with increasing the surfactant concentration in the fabrication of haloperidol-loaded PLGA nanoparticles [20]. Similar observations were noted in our case while fabricating RIS-loaded PLGA nanoparticles where an increase in surfactant of 0.025–0.5% resulted in a decrease in particle diameter from 123 nm to 109 nm, but at a very higher surfactant concentration (1%), the average particle size increased up to 228 nm.

The effect of varying polymer concentration and surfactant to polymer ratio of (D6 = 1:1.5, D7 = 1:2) showed that an increase in polymer concentration increased the particle size of NPs along with the percent EE, i.e., 205 nm (56% EE) and 294 nm (58% EE). Hence, the overall results suggest that polymer concentration in the organic phase is an important factor as it has a direct effect on particle size and EE. Research has reported that the PLGA contents in the organic phase vary between 56 mg and 100 mg and their influence on particle size was noted in such a way that it increases the average diameter from 205 nm to about 294 nm [19]. The effect of polymer concentration on particle size has been already evaluated by other authors for PLGA and PLA polymer [21,22,23]. The main reason for enlargement in particle size and EE is that higher polymer contents in the organic phase increase the viscosity, resulting in a poor dispersibility of polymer in the organic phase responsible for the formation of larger particles due to a poor emulsification process [21]. Similarly, as the high polymer contents increase viscosity, it results in a faster precipitation of the polymer and delays the drug diffusion across the polymer droplets responsible for higher encapsulation. For example, the EE was increased two times when the polymer concentration was increased from 20% to 32.5% by an investigator [24].

Similarly, in the case of drug content, as it increased from 0.05% to 0.1% (D4 formulation with a RIS to polymer ratio of 1:5.6 had a high drug content as compared to the other formulations), there was an in increase in the average particle diameter (168 nm) and a decrease in the percent EE (39%). A few investigators reported similar trends in the case of haloperidol-loaded PLGA nanoparticles [25] and praziquantel [19]. The effect of aqueous phase volume was also analyzed on particle size and %EE and it was noted that, as the volume of O: A increased up to 1:3 (D3 formulation), it had an average particle size of 130 nm with 20% EE. Doubling the volume of the aqueous phase has been reported to decrease the particle size and EE [25,26].

On the basis of minimum particle diameter (109 nm) and maximum EE (58%),the D2 formulation was selected for further evaluation and further optimized by varying the process parameters, such as sonication, the injection rate of organic phase and the stirring rate of aqueous media. Sonication decreased the particle diameter to 194 nm. The most important characteristic of nano formulations is their small particle size. The size is responsible for drug release and polymer degradation behavior [27], tissue penetration, bioadhesion [28] and intracellular uptake [29]. As risperidone’s major limitation is poor bioavailability and dose-related side effects, the selection of a smaller particle size of nanoparticles will improve not only its bioavailability, but also its dose will be decreased as compared to the actual dose. The polymer degradation rate is directly linked to the particle size. Nanoparticles with smaller size produce degradation products that diffuse to the surface easily, resulting in a slow degradation rate as compared to a larger particle size where the degradation rate is faster because of its larger size. The degradative products have a longer pathway to the surface of the particle resulting in the autocatalytic degradation of the remaining polymeric material [27], so the smaller-sized nanoparticles can sustain the drug release for a longer period of time.

The results show that the percentage of drug contents in NPs was in the range of 6–72%. The percentage yield was also determined and almost all formulations showed a good percent yield in the range of 18–40%, with the D2 formulation having 25% yield. It means that all the formulations prepared according to the precipitation method showed a good recovery of nanoparticles during the preparation.

The zeta potential or surface charge, being positive/negative, is a key parameter for the NP suspension stability. Particles with negative surface charge values repel each other and resist aggregation [30]. The zeta potential was found to be −9.3 ± 0.6 mv, where the negative charge is due to PLGA and carboxylic group as the zeta potential is less than 30 mv and not being equal to zero is acceptable [30].

The surface of PLGA NPs (blank and drug-loaded) evaluated through Scanning Electron Microscopy (SEM) showed an irregular spherical surface at a low resolution (Figure 7), but at a higher resolution of 50,000, it showed a smooth spherical surface topography. The particle size was also evaluated by SEM to confirm the size of NPs, where the slight increase in the size up to 300 nm could be due to the cryoprotectant used during the lyophilization process. The particle size and morphology of the nanoparticles play an important role in influencing the in vitro drug release, drug permeation as well as in vivo pharmacokinetics [31].

The in vitro release study revealed that a faster release (97% in 6 h) was observed from RIS suspensions as compared to NPs (57% in 6 h), reflecting a significant difference between their releases for 48 h with a *p*-value of 0.01 (*t*-test). The PLGA-NPs release profile showed a biphasic release pattern of initial burst release for 2 h, followed by a sustained release for 48 h. The release study was carried out for 72 h, but NPs showed a release of up to 93% in 48 h. This could be due to the faster diffusion of the drug from smaller particle sizes having a larger surface area for dissolution. Several drug release kinetics mechanisms, i.e., zero order, first order, Higuchi’s model and Hixson, were applied to the release profile of PLGA NPs, and the best fitness was obtained with the Korsmeyer–Peppas model, with an R^2^ value of 0.95 and an *n* value of 0.3, showing drug release from NPs to be effected through Fickian diffusion [32,33]. In the case of diffusion-controlled release mechanism, the drug molecules adsorbed on the surface of NPs showed a fast diffusion in the initial hours, followed by a slow diffusion from the core of particles in the second phase, as already discussed [34]. The other reason for the fast dissolution could be the grade of the polymer used for the nanoparticle preparation, as in our study, we used PLGA 50:50 grade resulting in a faster hydration of the polymer, subsequently boosting faster diffusion of the drug from polymer matrix, while for a more lipophilic drug as risperidone, PLGA with a grade higher than 50:50 would be suitable to achieve a prolonged release of several days, as investigated by [35].

Poloxamer lecithin organogel (PLO) has potential advantages over traditional bases in terms of drug release and absorption properties and skin permeation capabilities [17]. The prepared PLO gels had pH values in the range of 6.3–6.7, while our final optimized formulation had a pH of 6.7 near to neutral pH, which is more acceptable as a pH around 6 causes no irritation and discomfort to skin [36].

The results show that the formulations containing a maximum concentration of poloxamer and lecithin have a minimum T_gel_ due to more hydrophobic interactions among the polymer chains. The PLO4 formulation has more strength as compared to the other PLO formulations, which may be due to a high concentration of poloxamer (23%) and lecithin (20%). The gelation transition time of PLO was decreased by up to 30 s as compared to that of the P407 gel (without lecithin).

In vitro drug release studies for PLGA nanoparticles and NP-loaded PLO conducted for 72 h (Figure 6 and Figure 7) were compared with the release profiles of plain PLO (used as the control) showing significant differences in release patterns. The simple PLO showed a release up to 98% within 48 h, while NP-loaded PLO maintained its release at the control level for 72 h with a 77% drug release. As it was discussed earlier, PLGA NPs showed an initial burst release for 2 h; after that, they had a sustained release, but when these NPs were loaded into PLO with a high consistency, the burst release was retarded to some extent, such that during the first hour, the NP-loaded PLO release was only 10% as compared to the simple NPs, with 19% of the drug release in the first hour (Figure 7). At 9 h, the NP-loaded PLO release was 40% only, while the simple NP release was 64%, resulting in 1.5-fold decrease in burst release by loading PLGA NPs into PLO. So, the loading of NPs into PLO provides an additional barrier to its release due to the presence of a high concentration of poloxamer (23%) and lecithin (20%) forming a denser and more rigid fiber structure in the gel and decreased drug release. Previously [35], an antihypertensive agent Diltiazem hydrochloride (DZH) showed a decreased diffusion by using different concentrations of lecithin (20%, 30% and 40%) and pluronic (20%, 25% and 30%). The release kinetic mechanism applied on the PLGA NP-loaded organogel revealed that its release follows the Korsmeyer–Peppas model (Figure 8), with the highest coefficient value (R^2^ = 0.98, *n* = 0.3). So, the drug release mechanism for NPS and gel is diffusion.

Ex vivo permeation studies (Figure 9, Table 3) performed through full thickness rat skin for NP-loaded PLO and the control gel (PL407gel) showed a significant increase in permeation (*p* < 0.04; *t*-test). PLO showed a steady state flux of 82.67 ± 2.8 µg/cm^2^/h, while the control gel showed 31.53 ± 0.04 µg/cm^2^/h, which is three-fold higher than the control formulation in comparison, due to the interaction of lecithin with phospholipids of SC. The combination of poloxamer and lecithin provides a synergistic effect on drug permeation. The authors of [8] observed the enhanced penetration of methimazole in systemic circulation after a single topical application, revealing that it was due to the exfoliation and interaction of lecithin.

## 4. Conclusions

A novel optimized PLGA NP-loaded organogel encapsulating antipsychotic drug risperidone was developed successfully. The system combines the advantage of nanotechnology and microemulsion-based organogel (PLO) for the transdermal delivery of drug. To overcome the problems associated with the conventional dosage form, risperidone was encapsulated in nanoparticles by utilizing biodegradable polymer PLGA. PLGA nanoparticles were successfully fabricated with risperidone and showed positive results in terms of size, EE, surface charge, morphology and drug release. These NPs were successfully incorporated into the second carrier system (PLO), which had the optimum pH, strength, viscosity and gelation time to improve its release and enhance its permeation through skin barrier SC. The results confirm the improved drug release and several--fold increase permeation through the skin with a significance *p*-value less than 0.05. Hence, the investigated formulation could be a better alternative to the conventional route, enhancing patient compliance. Further studies including pharmacokinetics and biodistributionand biocompatibility studies will be performed to evaluate the potential of the PLGA NP-loaded organogel to be used as a drug delivery system.

## 5. Materials and Methods

### 5.1. Chemicals and Reagents

Risperidone was provided by Global Pharmaceuticals Islamabad, Pakistan; PLGA (RESOMER RG 502, MW 7000-17000), was purchased from Enovik Industries (Darmstadt, Germany); Isopropyl Myristate (Merck, Darmstadt, Germany), Poloxamer 407 (Pluronic F127 by Sigma life science, St. Louis, MO, USA) and soya lecithin (soya-phosphatidylcholine, Epikuron200) were purchased from BDH, West Yorkshire, UK.

Rat abdominal skin, freshly excised, was provided by The Animal Experimental Section of the Pharmacology Lab of the Department of Pharmacy QAU, Islamabad, Pakistan. (Approval No. #BEC-FBS-QAU2019-1258).

### 5.2. Drug Carrier Compatibility Studies

The drug to polymer interaction was analyzed using FTIR spectroscopy. The comparative FTIR spectra for RIS, poloxamer 407, drug–polymer physical mixture and final nanoparticle formulations in freeze-dried form were analyzed at a scanning range of 400–4000 cm^−1^ with a resolution of 4 cm^−1^.

### 5.3. Nanoparticle Fabrication

For the fabrication of biodegradable PLGA nanoparticles (blank and drug-loaded), the nano-precipitation technique [18] with little modifications was used. Briefly, a weighed amount of PLGA with a designated ratio between PLGA and drug (Table 1) was dissolved in 5 mL acetone at 45 °C with constant stirring until a clear solution was obtained. The volume of the organic phase was adjusted at room temperature (25 °C) due to the evaporation of solvent. Aqueous phase was prepared by dissolving P407 in 10 mL distilled water at a chill condition to obtain the concentration of 0.5% of poloxamer solution. Keeping the aqueous phase at a constant moderate stirring (600 rpm), the organic phase was subsequently added dropwise (@0.166 mL/min) at room temperature using a syringe needle of 23G. As a result of the evaporation of solvent, the polymer was precipitated in aqueous media and a turbid suspension was obtained. The remaining acetone and water were evaporated under vigorous stirring for 4–6 h. The prepared nanosuspension was centrifuged at 13,000 rpm for 25 min with double washing (with distilled water) to remove the unreacted polymer and the unbound drug, and the nanoparticle pellet was collected for characterization. The developed nanoparticles were finally lyophilized using 0.05% *w/v* mannitol as cryoprotectant. The obtained NPs were incubated at −20 °C in a freezer (Panasonic freezer) for 24 h and then lyophilized in a Freeze drier (Christ Alpha, Osterode am Harz, Germany) for 48 h to collect the solid nanoparticles.

### 5.4. Screening for Optimized RIS-Loaded PLGA Formulation

Several risperidone-loaded PLGA NP formulations were prepared and screened on the basis of different parameters by varying the drug to polymer ratio, organic phase to aqueous phase ratio and the surfactant to polymer ratio to achieve a suitable formulation having minimum particle size and optimum EE (Table 1). The selected screened formulation (D2) was subjected to further evaluation by changing process parameters, such as rate of injection, stirring rate and effect of sonication (Table 2).

### 5.5. Physicochemical Characterization of Nanoparticles

#### 5.5.1. Particle Size and Size Distribution of Nanoparticles

The particle size and size distribution (polydispersity index) of RIS-loaded PLGA nanoparticles were determined by using the DLS, dynamic light scattering, technique (Brookhaven instrument, New York, USA). For the DLS analysis, samples were prepared by dispersing produced nanoparticles suspension in an ultrapure water (5 times dilution for each sample) and added to a cuvette to analyze in triplicate (*n* = 3). The intensity of the scattered light indicated an average particle size and polydispersity index (PDI). PDI for samples was also analyzed by DLS to evaluate the homogeneity of nano-suspension. Values reported in the results are the mean value ± S.D.

#### 5.5.2. Zeta Potential Analysis

Zeta potential for PLGA nanoparticles was analyzed by Laser Doppler Anemometry (Malvern Zetasizer IV, Malvern Instruments Ltd., Malvern, UK). Samples were prepared in ultrapure water and added to disposable zeta cells to analyze at a 90° angle at room temperature (25 °C). For each sample, the mean value ± S.D. of three determinations was established.

### 5.6. Determination of Drug-Loading Parameters

The loading parameters evaluated for prepared RIS-loaded PLGA nanoparticles were entrapment efficiency, loading capacity and nanoparticle yield.

#### 5.6.1. Entrapment Efficiency

Entrapment efficiency was determined by indirect method. Briefly, the prepared nanoparticle suspension was centrifuged at 13,000 rpm for 25 min (3 times) to separate the supernatant containing free drug and unencapsulated polymer from the nanoparticle pellets. Samples were prepared by diluting a weighed aliquot of supernatant in methanol to make a proper dilution (10 times dilution) for UV-Visible spectrophotometry and analyzed at λmax 279 nm.

#### 5.6.2. Drug Loading

The nanoparticles containing pellet obtained after centrifugation were lyophilized and used for the direct drug-loading evaluation. The weighed lyophilized nanoparticles, i.e., 10 mg NPs, were dispersed in acetone to extract the drug from the nanoparticles. Acetone was evaporated by constant stirring for 2–3 h and then 5 mL methanol was added to solubilize the extracted drug in methanol. The sample was further diluted with methanol if required. Before analysis, samples were filtered through a syringe filter (0.2 µ) and analyzed at λmax 279 nm by using UV-Visible spectrophotometer.

### 5.7. Surface Morphological Analysis

The surface topography of nanoparticles was evaluated through SEM (Scanning electron microscopy, JSM 6400A, Tokyo, Japan) by a modified method reported by [37]. The sample was prepared by sprinkling lyophilized powder of NPs on a double-sided carbon conductive stub. To make the sample conductive, it was coated with Au. For the gold coating, ion sputtering device (JFC-1500, Joel., Tokyo, Japan) was used. The nanoparticles were viewed at an accelerating voltage of 15–20 kV.

### 5.8. Powder X-ray Diffractometery (PXRD) Analysis

The nature of the drug (crystalline or amorphous) in pure form and in the formulation was evaluated by Powder X-Ray Diffraction (Radiation source: Copper kα, STOE, Germany). The samples were prepared by filling lyophilized NP powder in an aluminum holder and analyzed. X-ray spectra were recorded using radiation Cu Kα, voltage 20 kV, and 5 mA generator current with monochromatic radiation and scintillation counter. The instrument was operated at a scanning speed of 0.5 s/step with a 2θ range of 5000–80,000 (begin and end, respectively).

### 5.9. In Vitro Drug Release Studies for NPs

#### Release Study for RIS-Loaded NPs

The RIS release study was carried out for PLGA NPs and simple RIS suspension (containing 3% theoretical drug loading) by adopting a method reported previously by [37] with slight modifications. A weighed quantity of risperidone-loaded PLGA NPs (35 mg freeze-dried powder having 3% theoretical drug loading) was dissolved in 5 mL of PBS (phosphate-buffered saline) of pH = 7.4, containing 0.025% (*w*/*v*) of Tween 80 to make a slurry. The slurry was placed in a dialysis bag and sealed with help of a thread. Cellulose acetate membrane with a molecular weight cut-off value of 12,000–14,000 was used as a dialysis membrane for donor compartment. The donor compartment was incubated in 70 mL of receptor medium-PBS with pH = 7.4 in a beaker to establish sink condition. The receptor compartment was maintained at a gentle shaking of 90 strokse/min at 37 °C in a water bath shaker. The same procedure was repeated for the preparation of donor and receptor compartments for simple drug suspension. At specific time intervals of 0, 1, 1.5, 2, 4, 6, 9, 24, 28, 48 and 72, hours, an aliquot of 3 mL was removed from the receptor compartment and restored with the same amount of fresh dissolution medium to maintain the sink condition. The amount of drug released was analyzed by using UV-Visible spectrophotometer at λ_max_ of 279 nm in triplicate and calculations were performed by using the calibration curve.

### 5.10. Preparation of PLGA NP-Loaded PLO

For the PLGA NP-loaded organogel, the previously optimized lecithin solution (20% *w/w* in IPM) and poloxamer P407 solution (23% *w*/*w* in D/Water) was prepared by the same method discussed earlier. Before mixing the organic and aqueous phases, a weighed quantity of lyophilized PLGA NPs (containing 3% theoretical drug loading) was added to the aqueous phases and uniformly dispersed with the help of a vertex mixer. Then, the oily phase was mixed with the aqueous phase in a ratio of 1:4; as a result, a beige-colored gel was formed.

### 5.11. Characterization of PLO

The different blank PLO formulations were characterized for organoleptic properties (color, odor, feel upon application and consistency) and pH [38].

#### 5.11.1. Determination of the Gelation Temperature (T_gel_) of PLO

The gelation temperature for PLO and polymeric poloxamer gel was evaluated by the modified tube inversion method [22]. According to this method, the difference between T_gel_ of PLO and poloxamer gel was compared and noted in triplicate. A glass assembly containing enough water placed on a hot plate was used. A thermometer (fisher scientific) was dipped in water to observe the temperature. The temperature of the assembly was set at 20 °C. Glass vials having a polymeric solution of poloxamer and PLO formulation were placed in an assembly such that the glass tube containing the gel was completely dipped in water. For each formulation, after 1 min, the tube was removed, inverted, checked for liquid flow and dipped again in water. The temperature of the water assembly was increased at a rate of 1 °C and gel formation was observed. The process was repeated until there was no flow, and this temperature was taken to be the gelation temperature of the gel.

#### 5.11.2. Determination of Viscosity, Strength and Gelation Transition Time

The viscosity of the final selected formulation was performed with a thermostatically controlled Brookfield programmable Rheometer (Model DV-1+ Brookfield viscometer) by using cylinder spindle 64 at 100 rpm and a temperature of 25 °C. The time required to convert sol into gel at a particular temperature was noted as gelation transition time. For gel strength, a previously reported method was used [39] with slight modifications. Gel strength is the time in which a glass assembly crosses the gel layer and reaches the bottom when it is placed over its surface. The gel strength was determined in seconds at a temperature of 34 °C ± 2 (skin temperature) for both combined and individual gel formulations.

### 5.12. In Vitro Drug Release Studies for PLO

In vitro release was conducted for optimized PLGA NP-loaded PLO (containing 3% theoretical drug loading) by using plain PLO (having the same amount of drug) as the control. This study was carried out by utilizing the same method adopted previously for RIS-loaded NPs in the section above. The average percent cumulative drug release versus time profiles was constructed to compare the drug release.

### 5.13. Determination of Drug Release Kinetics

To investigate the release kinetics mechanism, data obtained from in vitro drug release study of NPs and NP-loaded PLO were fitted in various kinetics models, i.e., zero order, fist order, Higuchi’s model, Korsmeyer–Peppas model and Hixson–Crowell model. The best fitness of these models in the form of correlation coefficient (R^2^) was used to describe the release patterns of the drug from NPS as well as NP-loaded PLO.

### 5.14. Ex Vivo Permeation Studies

Permeability studies were performed on rat skin. (Rat abdominal skin was provided by the Pharmacology Lab Department of Pharmacy, QAU Islamabad). For this purpose, hairs from excised abdominal skin area were removed with a razor and the skin was placed in a normal saline solution for an hour. The skin was evaluated visually for skin structure integrity before performing permeation studies. The ex vivo permeation study was performed using a horizontal Franz diffusion cell apparatus equipped with a magnetic stirrer and water jacket. The capacity of each Franz cell was 5.2 mL with a diffusional area of 0.77 cm^2^ maintained at 37 °C temperature. The drug permeated per cm^2^ of rat skin was calculated.

### 5.15. Statistical Analysis

All studies were replicated in triplicate (*n* = 3) and data are presented as mean ± S.D. The statistical analysis was conducted by using Microsoft Excel (version 2010). Student’s *t*-test and one-way analysis of variance (ANOVA) were performed on the in vitro studies data for comparison. A difference was considered statistically significant below the probability (*p*-value) level of 0.05. The software Sigma Plot (version 2012) was used to construct the patterns of in vitro release data. For the evaluation of drug release kinetics, the software DD Solver Ver. 1.0 was used.

## Figures and Tables

**Table 1:** Comparison of particle sizes and EE (%) of RIS-loaded PLGA NPs with different formulation ratios (*n* = 3).

F. code	RIS:Poly Ratio (*w*/*w*)	Surf:Poly Ratio (*w*/*w*)	O:A Ratio (*v*/*v*)	Particle Size (nm) Mean ± S.D.	EE % Mean ± S.D.	Drug Loading (%) Mean ± S.D.	NP Yield (%) Mean ± S.D.
D1	1:11.2	1:2.24	1:2	123.16 ± 10.27	35.87 ± 1.2	37.58 ± 0.08	40.0 ± 0.05
D2	1:11.2	1:1.1	1:2	109.48 ± 13.77	58.60 ± 0.89	54.68 ± 0.28	25.59 ± 1.2
D3	1:11.2	1:1.1	1:3	139.03 ± 17.5	20.56 ± 0.80	7.55 ± 0.03	24.65 ± 3.3
D4	1:5.6	1:1.1	1:2	168.09 ± 10.89	39.28 ± 0.82	30.49 ± 0.08	29.66 ± 5.1
D5	1:11.2	1.78:1	1:2	228.48 ± 3.315	47 ± 0.58	34.51 ± 0.48	18.02 ± 4.7
D6	1:11.2	1:1.5	1:2	205.66 ± 7.65	56.58 ± 0.2	45.89 ± 0.95	25.04 ± 6.2
D7	1:11.2	1:2	1:2	294.6 ± 24.21	58.45 ± 0.42	72.22 ± 0.66	46.95 ± 2.3

**Table 2:** Screening for process parameters and their effects (*n* = 3).

F. code	Injection Rate (mL/min)	Stirring Rate (rpm)	Sonication	P. Size (nm) Mean ± S.D.	EE % Mean ± S.D.	Drug Loading (%) Mean ± S.D.	NP Yield (%) Mean ± S.D.
D2a	0.166	100	No	206.94 ± 30.8	34.81 ± 0.42	48.14 ± 3.9	24.23 ± 4.1
D2b	0.66	600	No	209.01 ± 105.0	11.39 ± 0.22	6.11 ± 0.02	25.01 ± 0.6
D2c	0.66	100	No	225.78 ± 18.3	12.66 ± 0.16	19.68 ± 0.31	27.87 ± 0.8
D2d	0.166	1000	No	210.21 ± 5.5	11.69 ± 0.86	59.39 ± 0.77	23.12 ± 0.6
D2e	0.166	100	At 5 watts, 2 min	194 ± 7.7	41.21 ± 0.49	45.64 ± 0.06	25.11 ± 1.0

**Table 3:** Ex vivo permeation studies for NPs, NP-loaded PLO and PL407 gel.

Formulation Code	Flux (µg/cm^2^/h)	Permeability Coefficient Kp × 10^−3^ (cm^2^/h)	ER
PLGA NPs (P1)	12.7 ± 5.2	0.00634 ± 0.05	2.48
NP-loaded PLO (P2)	82.7 ± 2.8	0.05130 ± 0.08	2.60
Gel without lecithin (P3)	31.5 ± 0.04	0.01576 ± 0.06	_

## Data Availability

Data sharing not applicable.
